# Stigma and Its Impact on Glucose Control Among Youth With Diabetes: Protocol for a Canada-Wide Study

**DOI:** 10.2196/resprot.6629

**Published:** 2016-12-15

**Authors:** Anne-Sophie Brazeau, Meranda Nakhla, Michael Wright, Constadina Panagiotopoulos, Daniele Pacaud, Mélanie Henderson, Elham Rahme, Deborah Da Costa, Kaberi Dasgupta

**Affiliations:** ^1^ Department of Medicine McGill University Montreal, QC Canada; ^2^ Concordia University Montreal, QC Canada; ^3^ BC Children’s Hospital University of British Columbia Vancouver, BC Canada; ^4^ Alberta Children’s Hospital University of Calgary Calgary, AB Canada; ^5^ Centre Hospitalier Universitaire Sainte-Justine Université de Montréal Montreal, QC Canada

**Keywords:** type 1 diabetes, youth, stigma, perception, well-being

## Abstract

**Background:**

Stigma in chronic disease involves unwarranted rejection, judgement, or exclusion by others based on the chronic disease itself.

**Objective:**

We aim to determine the prevalence of stigma among youth and young adults with type 1 diabetes in Canada, to assess associations between stigma and glycemic control, and to explore ways to address stigma related to type 1 diabetes.

**Methods:**

The study includes 3 distinct phases: (1) refinement of survey questions, (2) assessment of test-retest reliability, and (3) a data collection and analysis phase (online survey and mailed-in capillary blood sample to assess hemoglobin A1c). A total of 380 youth and young adults (14 to 24 years old) with type 1 diabetes are being recruited through social media and clinic posters.

**Results:**

Phases 1 and 2 are complete, and phase 3 is in progress. Thirty participants completed phase 2. The survey includes the Barriers to Diabetes Adherence in adolescent scale (intraclass correlation [ICC]=0.967, 95% CI 0.931-0.984), the Self-Efficacy for Diabetes Self-Management measure (ICC=0.952, 95% CI 0.899-0.977), the World Health Organization-5 Well-Being Index (ICC=0.860, 95% CI 0.705-0.933), 12 closed-ended questions, and an additional 5 open-ended questions to explore challenges and solutions developed by the team of experts, including a patient representative.

**Conclusions:**

This will be the first large-scale survey to estimate the prevalence of stigma in young people with type 1 diabetes. The results of this study will allow for an appreciation of the magnitude of the problem and the need for developing and implementing solutions. This work is intended to provide an initial understanding of youth perspectives on the challenges of living with type 1 diabetes and will serve as a foundation for future research and action to help youth improve their experience of living with diabetes.

**Trial Registration:**

ClinicalTrials.gov NCT02796248, https://clinicaltrials.gov/ct2/show/NCT02796248 (Archived at http://www.webcitation.org/6mhenww3o).

## Introduction

Many youth with diabetes struggle with self-esteem, body image, social role definition, and peer-related issues [1]. During adolescence, peer relationships and acceptance by friends are essential [2]. In an effort to avoid being perceived as different by their peers, adolescents may engage in passive coping strategies such as withdrawal, avoidance of activities, and nonadherence to treatment regimens [3-5]. These behaviors may continue into early adulthood, now termed emerging adulthood. This period between the ages of 18 and 30 years is characterized by the challenges of establishing autonomy, personal identity, and making vocational and educational choices [6]. Emerging adults with diabetes must contend with complex developmental tasks while also dealing with their condition and its treatment [7].

Stigma related to chronic disease may be defined as, “negative social judgement based on a feature of a condition or its management that leads to perceived or experienced exclusion, rejection, blame, stereotyping and/or status loss” [8]. Adolescence and young adulthood are life stages that may be particularly vulnerable to stigma and its adverse impacts.

Canadian Chronic Disease Surveillance System data (2008/09) indicate that 20,492 children and adolescents (aged 10-19 years old) and 15,861 young adults (aged 20-24 years old) have diabetes [9]. Type 1 diabetes accounts for approximately 95% of diabetes in childhood and adolescence, and a large proportion of diabetes in emerging adults [10]. The universal and persistent need for insulin therapy in type 1 diabetes may compound the likelihood of stigma. Another reported cause of stigma is the loss of control resulting from hypoglycemia [11]. There is a paucity of evidence-based strategies to help people live with type 1 diabetes. Living with diabetes implies not only achieving control of blood glucose levels, but also feeling empowered, healthy, and happy. These factors have positive effects on quality of life [12]. Stigma may adversely impact diabetes management, mood, and a sense of well-being; longitudinal studies indicate that even mild emotional distress, a potential consequence of stigma, predicts *worse-than-expected* clinical and psychological outcomes [13]. Conversely, addressing stigma has the potential to enhance emotional states and health behaviors.

Stigma in diabetes has been understudied. Some recent investigations have examined stigma in adults with type 1 diabetes through in-depth interviews [8,14]. Such qualitative evaluations provide important insights into the causes and experiences of stigma, but cannot capture the prevalence of the problem. This issue is a key component that will be addressed in this study using a large, nation-wide sample.

The primary objective of this study is to determine the prevalence of stigma among youth and young adults with type 1 diabetes in Canada, through a nation-wide online survey. The survey includes a key subset of stigma-related questions used in a previous study [15]. In addition, in partnership with patient representatives and health care providers we selected, refined, and developed a set of both closed-ended and open-ended questions to more broadly capture the construct of stigma (sources, experiences, and consequences of stigma) and the challenges of living with type 1 diabetes. An innovative aspect of our study is that we are asking participants to mail in a capillary blood sample for assessment of glycemic control (hemoglobin A1c, a measure that captures overall glucose control over the prior 2-to-3 months [16]). Using these samples, in addition to the survey questions, we will assess the associations between stigma and hemoglobin A1c levels, hypoglycemia frequency, psychological well-being, and health behaviors (ie, potential consequences of stigma). Finally, by analyzing free text responses to open-ended questions, we will identify potential strategies to address stigma.

## Methods

All study procedures were reviewed and approved by the Institutional Review Board of the McGill University Health Centre. Electronic consent was obtained prior to completion of the survey.

### Study Design

**Figure 1:**
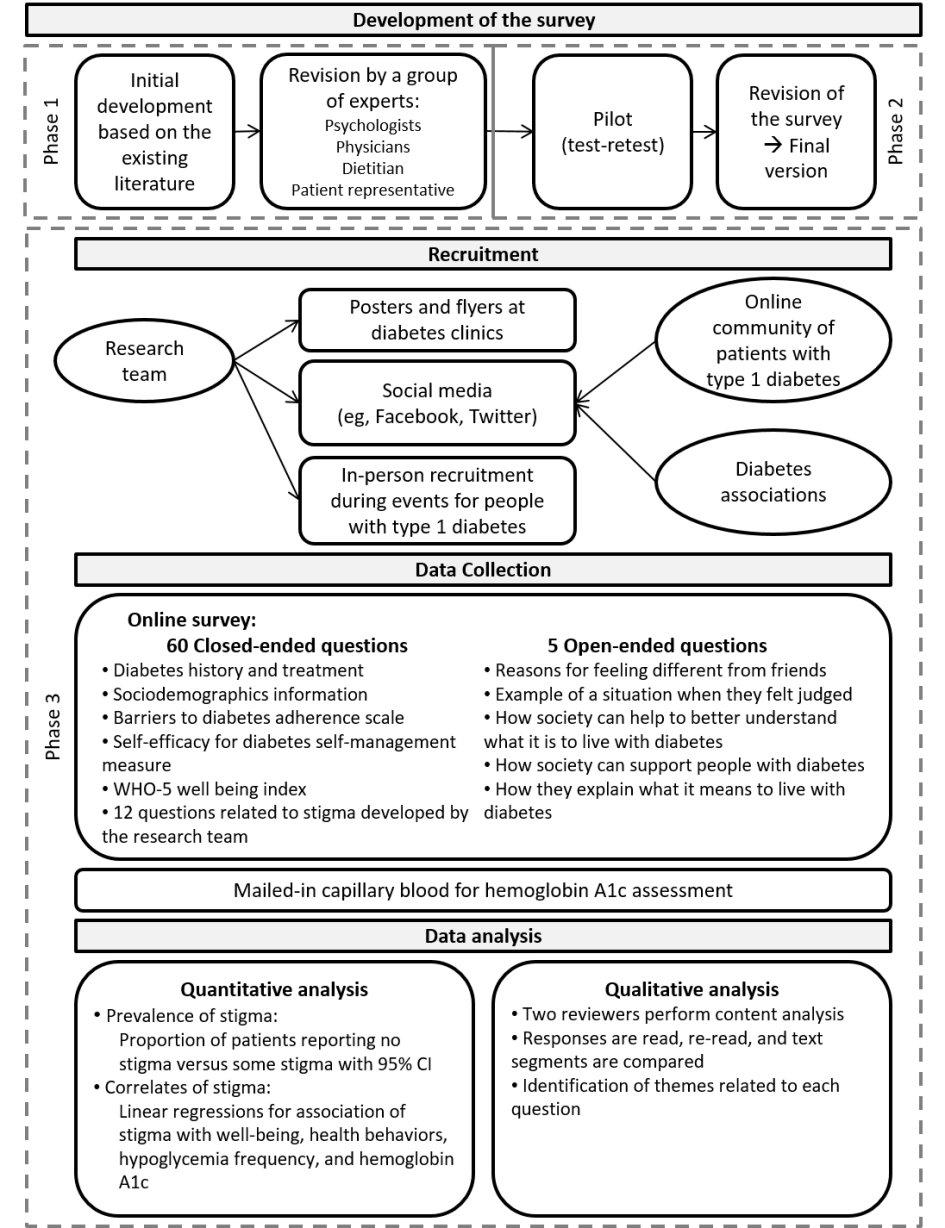
Study design.

This 1-year project is a cross-sectional online survey combined with mailed-in capillary blood samples for hemoglobin A1c assessment (Figure 1). The study includes 3 distinct phases: (1) survey question refinement and creation of an online questionnaire, (2) a pilot testing phase consisting of 30 participants completing the questionnaire on two separate occasions, and (3) a data collection phase (survey and hemoglobin A1c testing and analysis). Phases 1 and 2 have been completed.

#### Phase 1: Selection of Questions to Capture Stigma and Development of the Survey

A key set of questions included in our survey are derived from the Barriers to Diabetes Adherence (BDA) questionnaire developed by Mulvaney et al [15]. This 21-item tool includes 6 questions specifically addressing stigma, as well as 4 other aspects related to living with diabetes (stress and burnout, time pressure and planning, social support, and parental autonomy support). The BDA is reported to have good overall internal consistency (Cronbach alpha=.88) [15].

To gain a broader understanding of stigma in our specific population of interest, we included additional questions, guided by the revised framework for understanding diabetes-related stigma developed by Browne et al [8]. These investigators have highlighted the importance of addressing the causes, experiences, and consequences of stigma. *Causes* refer to the sources of stigma and features of the condition (ie, type 1 diabetes) and its management, *experiences* include judgment by others and stigmatizing practices, and *consequences* refer to the psychological, behavioral, and medical impact of diabetes-related stigma.

The questionnaire was developed by our research team, comprised of pediatric endocrinologists, clinician scientists, psychologists, a dietitian, and a patient representative. We conducted a survey of the literature and existing instruments, and developed our own questions.

The online questionnaire (French and English versions) was programmed into FluidSurveys [17], which is a low-cost online survey design tool used by researchers and government organizations. Data generated through FluidSurveys are stored in Canada. Participants can complete the survey directly online, or offline on tablets, laptops, or cell phones. The survey platform also allows participants to upload documents (eg, pictures, videos, or text). Participants can interrupt survey completion as needed, and continue at a more convenient time.

#### Phase 2: Pilot Testing

Thirty youth and young adults with type 1 diabetes were asked to complete the survey twice, spaced by one week, to assess test-retest reliability (see *Data Analyses*). Participants for the pilot phase were recruited through social networks (see *Recruitment*).

#### Phase 3: Data Collection

Following some adjustments to the survey tool based on the pilot phase, the final survey was programmed into FluidSurveys.

### Recruitment

Adolescents and emerging adults with type 1 diabetes aged 14-25 years are eligible for this study. Specifically, the study is publicized through posters at diabetes clinics, on Facebook pages, via Twitter messages, and on websites of diabetes organizations (eg, Canadian Diabetes Association, Diabète Québec). We also ask the organizations to email their members directly to inform them about the study, with a link embedded in the email message. We are also willing to be present at chapter meetings to provide information about the study. Flyers promoting the study are also posted at hospital centers in which study investigators are based. To further publicize the study, our patient representative (MW) communicates with potential participants through social networks for youth with type 1 diabetes.

Once participants have been directed to our website, they register to receive an emailed personal link to the survey. Upon completion of the survey, an Amazon.ca gift card is sent to them (Can $10). Participants are also asked if they are willing to mail in a capillary blood sample, as described below. When we receive the mailed-in sample, the participants are provided with a second gift card (Can $10).

### Glucose Control

Survey participants willing to mail in a capillary blood sample receive a kit for hemoglobin A1c testing (DTIL Laboratories, Inc., Thomasville, GA, USA) with a prepaid envelope for mailing the sample back to Montreal (QC, Canada). The kit includes a sample vial that contains a preservative (ethylenediaminetetraacetic acid), a vial holder, a single use lancet, a capillary tube device to draw up a small amount of blood after lancing, and a Ziploc bag. We batch ship the samples to a laboratory in the United States. The AccuBase A1c Test Kit is a nonfasting, finger stick, whole blood mail-in test requiring a very small blood volume (0.001 milliliters). Upon collection, samples are stable for 45 days without refrigeration. Samples are analyzed using a two-step process: the screening step detects hemoglobin variants and/or disturbed erythrocyte kinetics by ion-exchange high performance liquid chromatography; the second step includes the use of an interference-free procedure (high performance liquid chromatography-boronate affinity) that provides a hemoglobin A1c value free of possible interferences, including chemically modified derivatives [18].

### Sample Size

To date, no data exists regarding the prevalence of stigma in adolescents and emerging adults with type 1 diabetes. We estimate a 50% prevalence for sample size calculations, as this is the proportion that mandates the largest sample size. Given that approximately 34,535 adolescents and emerging adults in Canada have type 1 diabetes (95% of 36,353 diabetic individuals [9]), to detect a 50% proportion with stigma to an accuracy of 5% would require 380 survey completers (two-sided 95% CI for a single proportion with normal approximation, using nQuery Advisor 7.0). Based on our previous experience [19], we estimate that 52% of the respondents will complete the mailed-in hemoglobin A1c test. Thus, a subsample of 200 participants will provide objective data on hemoglobin A1c levels.

### Data Analyses

#### Development and Pretesting

The test-retest reproducibility of the scales was determined by comparing results on the two separate occasions that the questionnaire was completed (30 participants). Test-retest reliability was examined using single-measure intraclass correlation coefficients (ICC) and 95% CIs, with ICC <0.40 indicating poor agreement, 0.40-0.74 indicating fair to good agreement, and >0.75 indicating excellent agreement [20].

#### Participant Characteristics

Means and standard deviations (SDs), medians and interquartile ranges, or proportions are being used (as appropriate) to report participant characteristics.

#### Prevalence of Stigma

For the primary analysis, we will evaluate the responses to the 6 questions addressing stigma in the BDA questionnaire [15]. Each of the items involves a 1-to-5 Likert scale. For our purposes, *no stigma* will be a score of 6 out of a potential 30. We will determine the proportion of individuals with a score >6 and the corresponding 95% CI. We will then examine individual questions addressing stigma through our questionnaire, selecting a cutoff score tailored to each question. Our data will give us the ability to estimate an overall prevalence of diabetes-related stigma among youth and young adults with type 1 diabetes. We will then estimate the prevalence of stigma in subgroups (ie, boys, girls, ethnocultural groups, teens, and young adults)

#### Correlates of Stigma

Linear regression will be used to evaluate associations of stigma with psychological well-being, health behaviors, hypoglycemia frequency, and hemoglobin A1c, with additional potential predictors included in the models (eg, age, sex, sexual orientation/gender identification, ethnocultural background).

#### Analyses of Responses to Open-Ended Questions

Two reviewers will perform qualitative content analysis of responses to open-ended questions (one of whom is a patient representative). The responses will be read twice, and text segments will be compared, seeking similar or repeated ideas. The final step will involve labeling identified themes for each question. We have employed a similar analytical strategy for qualitative studies in diabetes [21,22].

## Results

### Phase 1

In addition to the BDA, we considered 2 additional questions developed by Folias et al to capture stigma (as reported in an American Diabetes Association abstract [23]), as well as other questions related to lived experiences with type 1 diabetes and managing young people with type 1 diabetes (eg, stigma within social media networks) [24].

In addition to stigma, we query peer support, quality of life and well-being, diabetes history and current treatment, and socio-demographic information. We considered several existing tools and instruments, such as the Hypoglycemia Patient Questionnaire [25], the Self-Efficacy for Diabetes Self-Management measure (SEDM; 10 items, test-retest ICC=0.89, Cronbach alpha=.90 [26]), the Problem Areas in Diabetes Scale (a 20-item measure assessing feelings related to living with diabetes and its treatment, including guilt, anger, frustration, depressed mood, worry, and fear; Cronbach alpha=.92 [27]), the Pediatric Quality of Life Inventory Generic Core Scales and Diabetes Module (a 28-item questionnaire, age-specific for 13-18 year-olds; Cronbach alpha=.88 [28]) and the World Health Organization (WHO)-5 Well-Being Index, (a validated 5-item questionnaire assessing subjective psychological well-being [29]).

Demographic factors including age, sex, sexual orientation/gender identification, and ethnocultural background are queried to ascertain the prevalence of diabetes-related stigma in different demographic subgroups. Owing to their potentially sensitive nature, however, the questions regarding sexual orientation/gender orientation are explicitly optional [30]. Following completion of closed-ended questions, participants respond to open-ended questions that seek to capture experiences and perceptions of stigma, as well as ideas about how stigma may be effectively addressed. Participants are permitted to upload explanatory materials (eg, videos, testimonials, pictures, drawings).

The survey that underwent pilot testing included 7 socio-demographic questions, 5 questions regarding diabetes history and treatment, the BDA scale, the SEDM scale, and the WHO-5 Well Being Index, as well as 12 questions related to stigma developed by the team (eg, *reasons for feeling judged*, *by whom they feel being judged the most*) and an additional 4 open-ended questions (eg, *describe a situation in which you felt judged*, *how can society can better understand what it is like to live with type 1 diabetes?*).

### Phase 2

The mean age of the 30 participants in the pilot phase was 20 years, and type 1 diabetes duration averaged approximately 9 years (Table 1). A large majority of participants were female, and half used multiple daily injections of insulin, while half were on insulin pump therapy (Table 1). The mean hemoglobin A1c value was slightly above target (ie, 7.0% [16]) at 7.6%, and the average number of hypoglycemic episodes in the prior week was approximately 3 (Table 1).

**Table 1:** Participants’ characteristics.

Characteristics		Mean (SD) or frequency (%)
Age, years		20.5 (2.8)
Age at diagnosis, years		11.3 (5.5)
Diabetes duration, years		9.2 (5.1)
**Sex**		
	Female	24 (80%)
	Male	6 (20%)
**Language**		
	English	19 (63%)
	French	11 (37%)
Last hemoglobin A1c, %		7.6 (1.4)
**Insulin treatment**		
	Multiple daily injections	15 (50%)
	Continuous subcutaneous insulin injection (insulin pump)	15 (50%)
Number of hypoglycemic events in the previous week		3.1 (2.6)

In the pilot phase, the average time between the two completions of the survey was 9.8 days (SD 4.5). A mean of 20:19 minutes (SD 8:52) were required to complete the survey. ICCs were high for the three scales included in the pilot version of the survey (BDA ICC=0.967, 95% CI 0.931-0.984; SEDM ICC=0.952, 95% CI 0.899-0.977; and WHO-5 Well Being Index ICC=0.860, 95% CI 0.705-0.933). Following the analyses of the pilot phase, minor modifications were made to the survey. The final version includes 60 closed-ended questions and five open-ended questions. Data collection is in progress.

## Discussion

Our study will be the first nation-wide evaluation of stigma in youth and young adults with type 1 diabetes. Previous studies have largely been qualitative; our investigation will include qualitative components but will also permit an estimate of prevalence. This strategy will allow for an appreciation of the magnitude and scope of the problem, and the development of strategies to help patients live better lives. Novel aspects of our methodology include (1) the use of an online survey, permitting evaluation across a large geographic area and inclusion of many individuals; (2) incorporation of a mailed-in capillary blood sample, allowing for objective assessments of glycemic control; (3) use of social media to enhance recruitment; and (4) inclusion of a patient representative on the research team, which provides an expert on the experience of living with type 1 diabetes. The questionnaire has been developed and pilot tested, and demonstrates good test-retest reproducibility. Our work is intended to provide an initial understanding of youths’ perspectives on difficulties and potential solutions for a normal life, and to serve as foundation for future research that helps people with type 1 diabetes achieve a good quality of life.

Online surveys are useful tools for achieving wide geographic coverage (which are particularly useful in large countries like Canada) and a large sample size, at relatively low cost. A survey-based approach in a private location may be preferable to interviews and/or witnessed survey completion when dealing with sensitive topics [31]. We have used online surveys as part of a prospective study examining depressive symptoms in 622 men (mean age 34.3 years, SD 5.0) during the first postnatal year with a newborn [32]. Participants completed standardized online self-report questionnaires measuring depressed mood, physical activity, sleep quality, social support, marital adjustment, life events, financial stress, and demographic factors during their partners’ third trimester of pregnancy. We determined that >10% of men experience depressed mood in the first year after their child’s birth [32]. We have adopted a similar method in the current study to capture the concept of stigma. Stigmatizing practice can come from family members, friends, health care providers, classmates, teachers, colleagues, and employers, among others. A self-report method, rather than interviews, was considered an ideal method to question this young population due to the high level of privacy that this approach affords.

We hypothesize that prevalence of stigma will be high in this age group, and may negatively impact diabetes management, as reflected through hemoglobin A1c (ie, high values reflect high blood glucose) and frequency of hypoglycemia episodes. To measure hemoglobin A1c, we are using mailed-in capillary blood samples. In a previous study, we used mailed-in capillary blood samples to measure glucose levels, in an effort to determine the prevalence of diagnosed and undiagnosed diabetes in the province of Quebec [19]. In that study, 52.2% (954/1829) of survey respondents provided mailed-in blood samples, of which approximately 90% were analyzable [19]. To our knowledge, no previous study has specifically examined the relationship between stigma and diabetes control in type 1 diabetes, although positive associations between emotional distress and prolonged suboptimal glycemic control have been reported [33]. This study will allow us to determine whether important medical consequences are associated with stigma (ie, an association between stigma and high hemoglobin A1c levels and/or hypoglycemia frequency).

Our previous studies have largely relied on in-person recruitment, even when the survey instruments are online (eg, introductions by clinic staff, clinic posters). However, the present study has largely relied on online and social media-based recruitment strategies. We have contacted many small diabetes organizations that focus on youth and young adults with type 1 diabetes. Many of these organizations are local initiatives that have generously tweeted about our study or posted it on their Facebook pages. This recruitment strategy appears to be particularly effective in this young, social media-savvy patient population.

Our team includes a patient representative, which is critical to the relevance of this study. There is an increasing recognition of the importance of patient representatives in health care research, due to their insights into defining research questions, adapting tools, interpreting results, and participating in knowledge translation efforts [34]. Patients are recognized as experts in their experience of disease. Our team includes such a representative, who has participated in all aspects of this study to date, and is remunerated for his work. This representative was instrumental in the selection of questions that were included in the survey, and assisted with participant recruitment via social media. This team member is also participating in the identification of themes by reviewing the responses to our open-ended questions.

Limitations of our study include potential selection bias; those who opt to participate may experience more (or less) stigma than others with type 1 diabetes, and may therefore be more likely to complete the survey. Recruitment in general is challenging, as even online surveys may be perceived as burdensome. Additionally, some participants may be reluctant to mail in capillary blood samples, even though this patient population is accustomed to capillary blood sampling for self-monitoring purposes. Our study will not capture the directly-reported perspectives of family members or friends, although we may address these factors in a future study.

Regarding future research directions, we are exploring the possibility of developing a more comprehensive stigma scale for this population. We plan to use the information from the present study to design a follow-up survey, similar to the scale developed for type 2 diabetes [35]. Such a tool could permit evaluation of trends and changes over time, with potential for incorporation into national surveys such as the biennial Canadian Community Health Survey [36].

Our study will highlight the importance of stigma in the day-to-day lives of young people with type 1 diabetes, as well as its association with diabetes control and adherence to treatment in a Canadian population. Stigma is a social construct, and therefore differs widely across societies [37]. It is important to gather information reflective of various populations. Patient-derived potential solutions will be formulated to reduce stigma in this clinical population.

## Abbreviations

BDA: Barriers to Diabetes Adherence
CI: confidence interval
FRQS: Fonds de recherche du Québec-Santé
ICC: intraclass correlation
SD: standard deviation
SEDM: Self-Efficacy for Diabetes Self-Management
WHO: World Health Organization
